# Integrated Trinity Test With RPA-CRISPR/Cas12a-Fluorescence for Real-Time Detection of Respiratory Syncytial Virus A or B

**DOI:** 10.3389/fmicb.2022.819931

**Published:** 2022-03-31

**Authors:** Ling Gong, Xiaowen Wang, Zhu Li, Guichuan Huang, Wei Zhang, Jin Nie, Chunyan Wu, Daishun Liu

**Affiliations:** ^1^The First Clinical Medical College, Jinan University, Guangzhou, China; ^2^Department of Respiratory Medicine, The Third Affiliated Hospital of Zunyi Medical University, The First People’s Hospital of Zunyi, Zunyi, China; ^3^Department of Respiratory Medicine, The Affiliated Hospital of Zunyi Medical University, Zunyi, China; ^4^Department of Basic Medicine, Zunyi Medical University, Zunyi, China

**Keywords:** respiratory syncytial virus A or B, RPA, CRISPR/Cas12a, fluorescence, real-time detection

## Abstract

Respiratory syncytial virus (RSV) is a common virus that causes respiratory infection, especially severe respiratory infection in infants and young children, the elderly people over 65 years old, and people with weak immunity. Currently, RSV infection has no effective vaccine and antiviral treatment. The number of deaths due to RSV infection increases every year. Moreover, RSV A infection occurs in a large number and has severe clinical symptoms and complications than RSV B infection. Therefore, the development of a simple, rapid, and inexpensive detection method with high amplification efficiency, high sensitivity, and specificity is very important for the diagnosis of RSV A or RSV B infection, which can help in the early clinical medication and prevent the progress of the disease. Therefore, we developed an integrated trinity test with an RPA-CRISPR/Cas12a-fluorescence (termed IT-RAISE) assay system to detect RSV A or RSV B. The characteristic of the IT-RAISE system is that after target recognition, the reporter single-stranded DNA (ssDNA) is cleaved by Cas12a that is activated by different crRNAs to detect the generated fluorescent signal. This method is simple and helps in adding all reagents rapidly. It is a high-sensitive method that can detect 1.38 × 10^1^ copies/μl of the target sequences, and it can distinguish RSV A or RSV B infection within 37 min. In addition, clinical specimens were detected for IT-RAISE system. It was found that the sensitivity and specificity of RSV A were 73.08 and 90%, respectively, and those of RSV B were 42.86 and 93.33%, respectively. The cost of ONE specimen for IT-RAISE system was approximately $ 2.6 (excluding rapid RNA extraction and reverse transcription costs). IT-RAISE system has good clinical application prospects for detecting RSV A or RSV B infection; it is a simple, rapid, and inexpensive method with high amplification efficiency, high sensitivity, and high specificity. The IT-RAISE system might also detect other viral or bacterial infections.

## Introduction

Respiratory syncytial virus (RSV) is a single-stranded RNA negative-sense virus with a filamentous envelope (Battles and McLellan, 2019; Gong et al., 2021) and includes two antigenic subtypes A and B (Mejias et al., 2017). In infants and young children, the virus commonly triggers lower respiratory tract infections, which include bronchiolitis and pneumonia, and occurs in approximately 60% of preschool children worldwide (Mejias et al., 2017). The virus is also an important pathogen in the elderly (≥65 years) and those with weak immunity (Pierangeli et al., 2018). According to World Health Organization data, approximately 33 million new infections occur each year globally, with more than 3.4 million severely infected individuals who require hospitalization (Atherton et al., 2019; Kirolos et al., 2019). By combining hospital and community death data, approximately 94,600–149,400 children under the age of 5 years are estimated to die worldwide each year (Barr et al., 2019). RSV is a ubiquitous infection with a significant clinical and financial burdens (Barr et al., 2019). A large number of RSV A infections occur, with more severe clinical symptoms and complications than RSV B (Panayiotou et al., 2014; Agoti et al., 2015; Calderón et al., 2017; Abou-El-Hassan et al., 2019; Yu et al., 2019). Therefore, early viral detection could improve RSV A or RSV B infection treatments and its related complications.

Respiratory syncytial virus detection methods include viral culture, direct fluorescent antibody detection, rapid antigen detection, high-throughput sequencing, rapid molecular detection, and multiple molecular detection (Nam and Ison, 2019). At present, the most common method is rapid molecular detection and includes the following: RT-PCR assay, GeneXpert Xpress Flu/RSV (Xpert; Cepheid, Sunnyvale, CA, United States; Arbefeville et al., 2017), BioFire FilmArray respiratory panel (RP; BioFire Diagnostics, Salt Lake City, UT, United States), Diasorin Simplexa Flu A/B and RSV (Simplexa; Diasorin Molecular, Cypress, CA, United States; Hindiyeh et al., 2013), Aries Flu A/B and RSV (Luminex Corporation, Austin, TX, United States; Juretschko et al., 2017), Cobas influenza A/B and RSV (Liat; Roche Diagnostics, Indianapolis, IN, United States; Binnicker et al., 2015), and Panther Fusion Flu A/B and RSV (Fusion; Hologic Inc., San Diego, CA, United States). Of these methods, RT-PCR normally includes a reverse transcription (RT) and amplification protocol with a simple reagent mix and easy to interpret results; however, it requires a considerable manual input and takes approximately 3 h for results; GeneXpert Xpress Flu/RSV requires repeat testing, BioFire FilmArray respiratory panel and Diasorin Simplexa Flu A/B and RSV assay are time-consuming and exhibit low sensitivity, Aries Flu A/B and RSV, Cobas influenza A/B and RSV, and Panther Fusion Flu A/B and RSV are time-consuming and require repeat testing (Banerjee et al., 2018; Banerjee et al., 2019; Zou et al., 2019).

For rapid molecular testing, although the GeneXpert Xpress Flu/RSV kit rapidly detects RSV within 30 min and has a high positive rate, it is impossible to distinguish between RSV A and RSV B infections. Several methods can distinguish RSV A or RSV B infection, which include eSensor Respiratory Viral Panel, ePlex Respiratory Pathogen Panel, Verigene Respiratory Virus Plus Nucleic Acid Test, Verigene RP Flex, and Nx-TAG Respiratory Virus Panel, but the methods are time-consuming (Nam and Ison, 2019). The eSensor Respiratory Viral Panel assay requires 8 h and the amplified products require manipulation, the ePlex Respiratory Pathogen Panel assay requires 2 h, the Verigene Respiratory Virus Plus Nucleic Acid assay requires 3.5 h, the Verigene RP Flex assay requires 3.5 h, and the Nx-TAG Respiratory Virus Panel assay requires 4–8 h (Nam and Ison, 2019).

To address these issues, we developed a diagnostic method using an RPA-CRISPR/Cas12a-fluorescence assay system. CRISPR is advantageous as it is cheap, easy, and rapid. Several studies have reported that a combination of LbCas12a-CRISPR RNA (crRNA) complex and complementary single- or double-stranded DNA releases a strong non-specific single-stranded DNA (ssDNA) with both *cis-* and *trans*-cleavage activities (Gootenberg et al., 2018; Li et al., 2018; Strecker et al., 2019), which promote the Cas12a as a novel gene detection and imaging tool. Currently, to increase sensitivity and specificity of the CRISPR/Cas diagnostic system, a specific amplification method such as recombinase polymerase amplification (RPA) is used for target sequence amplification (Yuan et al., 2020). Chen et al. (2018) observed that RPA and CRISPR/Cas12a *trans*-cleavage activity (or DETECTR) applications distinguished between human papillomavirus types 16 and 18 in human specimens. RPA mainly relies on its recombinase capability to bind single-stranded nucleic acids, ssDNA-binding proteins, and strand-displacement DNA polymerase (Ding et al., 2020). A major advantage of RPA is that it amplifies specific nucleic acid sequences at 37–42^°^C, with an RPA product readout within 20–30 min (Xi et al., 2019; Xu et al., 2020). The main fluorescence assay principle involves the fluorescent labeling of FAM and the quencher, TAMRA at 5′ and 3′ ends, respectively. A fluorescence microplate reader or a real-time fluorescence PCR machine is used for rapid data reading and is widely used in hospital laboratories. Therefore, an RPA-CRISPR/Cas12a-based fluorescence assay system could be a simple, rapid, inexpensive, and alternative approach to detect RSV A or RSV B with high amplification efficiency, high sensitivity, and high specificity.

**FIGURE 1:**
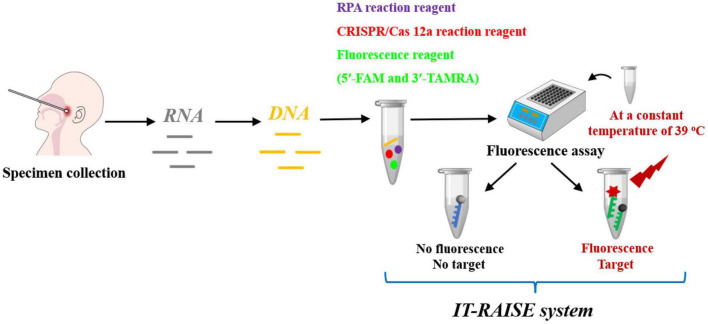
The working principles of the IT-RAISE system for RSV A or RSV B detection. If RSV A or RSV B is present, Cas12a cleaves RSV A or RSV B RPA products and a fluorescent single-stranded DNA (ssDNA) reporter (5′-FAM and 3′-TAMRA). Fluorescence quenching is terminated and fluorescence appears. If RSV A or RSV B sequences are not present, fluorescence quenching occurs and no fluorescence appears.

In this study, we first identified specific RSV A and RSV B DNA sequences, established a detection system using RPA, Cas12a, and a fluorescence assay step-by-step approach, and verified whether system could efficiently detect RSV A or RSV B (Supplementary Figure 1A). The method showed great promise for RSV A or RSV B practical applications. Furthermore, to save more time and simplify operating processes, we used a one step approach to develop an integrated trinity test with an RPA-CRISPR/Cas12a-fluorescence (termed IT-RAISE) system for RSV A or RSV B detection based on the step-by-step experiment (Supplementary Figure 1B). In short, all reaction reagents and target DNA sequences were combined and verified and showed the system functioned well. Finally, we used clinical oropharyngeal swab specimens to verify whether our IT-RAISE system efficiently detected RSV A or RSV B (Figure 1). The system detection principle is based on the fact that when no RSV A or RSV B sequences are present, a state of fluorescence quenching occurs and no fluorescence appears. If RSV A or RSV B sequences are present, they will be cleaved by Cas12a, fluorescence quenching is terminated, and fluorescence appears. Finally, we distinguished between RSV A or RSV B infections using Cas12a to cleave target sequences: the process was simple, rapid and demonstrated high amplification efficiency, high sensitivity, and high specificity.

## Materials and Methods

### Main Materials and Reagents

Specific RSV A- and RSV B sequences were obtained from Sangon Biotech (Shanghai, China; Table 1). RPA primers were obtained from Zhongke Yutong Biotechnology (Shanxi, China; Table 1). crRNAs, ssDNA reporter, and ssDNA-Cy5 were obtained from ExonanoRNA (Guangdong, China; Table 1). EnGen^®^ Lba Cas12a (Cpf1) enzymes were obtained from New England Biolabs (New England, United States). TwistAmp™ Liquid Basic kit was purchased from TwistDx™ Limited (Cambridge, United Kingdom). pUC57 plasmids were obtained from Sangon Biotech (Shanghai, China). Human bronchial epithelial cell line (BEAS-2B; Cat # CRL-9609) and RSV A standard strain (Long; Cat #ATCC VR-26) were purchased from American Type Culture Collection (ATCC, United States). An RSV nucleic acid PCR fluorescent probe kit was purchased from Daan Gene Company (Guangdong, China). Disposable samplers with virus preservation solution (COPAN flocked swabs) were purchased from Yongkang Company (Hebei, China). A Universal RT-PCR kit was purchased from Solarbio Company (Beijing, China). Virus RNA extraction kit in 10 min was purchased from Baiao Laibo Company (no. MT0012, Beijing, China). PrimeScript RT Master Mix was purchased from Takara Company (no. RR036A, Beijing, China).

**TABLE 1:** Oligonucleotide sequences used in this study^a–f^.

Oligonucleotide	sequence (5′–3′)
ssDNA reporter	FAM-TTTTTTTTATT-TAMRA
RSV A DNA sequence	AAGCTTTAGATAGGATTGATGAAAAATTAAGTGAAATACTAGGAATGCTTCACACATTAGTAGTGGCAAGTGCAGGACCTACATCTGCTCGGGAT GGTATAAGAGATGCCATGGTTGGTTTAAGAGAAGAAATGATAGAAAAAATCAGAACTGAAGCATTAATGACCAATGACAGATTAGAAGCTATGGC AAGACTCAGGAATGAGGAAAGTGAAAAGATGGCAAAAGACACATCAGATGAAGTGTCTCTCAATCCAACATCAGAGAAATTGAACAACCTATTG GAAGGGAATGATAGTGACAATGATCTATCACTTGAAGATTTCTGATTAGTTACCAATCTTCACATCAACACACAATACCAACAGAAGACCAACAA ACTAACCAACCCAATCATCCAACCAAACGAATTC
RSV B DNA sequence	AAGCTTCTAGATAGAATTGATGAAAAATTAAGTGAAATATTAGGAATGCTCCATACATTAGTAGTTGCAAGTGCAGGACCCACTTCAGCTCGCGAT GGAATAAGAGATGCTATGGTTGGTCTGAGAGAAGAAATGATAGAAAAAATAAGAGCGGAAGCATTAATGACCAATGATAGGTTAGAGGCTATGG CAAGACTTAGGAATGAGGAAAGCGAAAAAATGGCAAAAGACACCTCAGATGAAGTGCCTCTTAATCCAACTTCCAAAAAATTGAGTGACTTGTT GGAAGACAACGATAGTGACAATGATCTGTCACTTGATGATTTTTGATCAGTGATCAACTCACTCAGCAATCAACAACATCAATAAAACAGACATC AATCCATTGAATCAACTGCCAGACCGAAGAATTC
RSV A-TS1	UAAUUUCUACUAAGUGUAGAUGAAGUUCACUAUCUAGUAAC
RSV A-TS2	UAAUUUCUACUAAGUGUAGAUUGAUUAGUUACCAAUCUUCA
RSV B-TS1	UAAUUUCUACUAAGUGUAGAUGUAGUUCACUGUCUAGUAAC
RSV B-TS2	UAAUUUCUACUAAGUGUAGAUAUCAGUGAUCAACUCACUCA
RSV B-TS3	UAAUUUCUACUAAGUGUAGAUAUCAGUGAUCAACUCACUCAGC
RSV B-TS4	UAAUUUCUACUAAGUGUAGAUAUGAUUUUUGAUCAGUGAUC
RSV A-RPA-F (110 bp)	GAGAAATTGAACAACCTATTGGAAGGGAATG
RSV A-RPA-R	GTCTTCTGTTGGTATTGTGTGTTGATGTGA
RSV B-RPA-F (122 bp)	TAATCCAACTTCCAAAAAATTGAGTGACTTG
RSV B-RPA-R	TGTTTTATTGATGTTGTTGATTGCTGAGTG
RSV A activator	TCACTGTTACTAGATAGTGAACTTCTAAAGACTAATCAATGCATTGATTAGTCTTTAGAAGTTCACTATCTAGTAACAGTGA
RSV B activator	CACTTGATGATTTTTGATCAGTGATCAACTCACTCAGCAATCGATTGCTGAGTGAGTTGATCACTGATAAAAATCATCAAGTG
RSV A–F (100 bp)	ACTGCAATCAYACAAGATGCAACRA
RSV A–R	CAGATTGRAGAAGCTGATTCCA
RSV B–F (100 bp)	ACTTACCTTACTCAAGTCTCACCAGAAA
RSV B–R	TTGTRGCTGARTTTGTGTGGAT

*^a^FAM and TAMRA are fluorescence and quencher labeled at 5′–3′ ends.*

*^b^RSV A-TS1 and RSV A-TS2 are selected crRNAs for RSV A. TS is target sequence.*

*^c^RSV B-TS1, RSV B-TS2, RSV B-TS3, and RSV B-TS4 are selected crRNAs for RSV B.*

*^d^RSV A and RSV B RPA are designed according to the specific RSV A and RSV B sequences on scribed part.*

*^e^RSV A and RSV B activators are synthetic fragments.*

*^f^RSV A and RSV B are PCR primers used to amplify clinical specimens and for Sanger sequencing.*

### Target DNA Design, Cloning, and Plasmid Dilutions

Before the study commencement, we performed multiple sequence alignments on all RSV A and RSV B genomes in GeneBank. Using the NCBI-BLAST search tool, we determined specific RSV A or RSV B DNA sequences (Table 1). The RPA primers for specific RSV A and RSV B DNA sequences were designed using Premier Biosoft designer (Table 1), confirmed by NCBI-BLAST, and referred to TwistAmp Assay Design Manual principles. The RPA primers were designed to pick up all RSV strains. To test RPA stability of amplification effects, target viral DNA fragments were individually integrated into pUC57 plasmids, serially diluted, and the lowest target sequence concentration detected. About 4 μg of plasmid lyophilized powder was centrifuged at 10,000 rpm for 2 min and 100 μl of ddH_2_O added and mixed (concentration = 40 ng/μl/tube). The mixture was divided into five tubes, 20 μl each tube; 1 μl contains 1.38 × 10^8^ copies of plasmid. Then, 180 μl of ddH_2_O was added into 20 μl mixture (concentration = 4 ng/μl/tube); 1 μl contains 1.38 × 10^7^ copies of plasmid. The mixture was serially diluted 10-folds in ddH_2_O each time, with 1 μl finally containing 1.38 × 10^0^ copies of plasmid.

### Recombinase Polymerase Amplification

**FIGURE 2:**
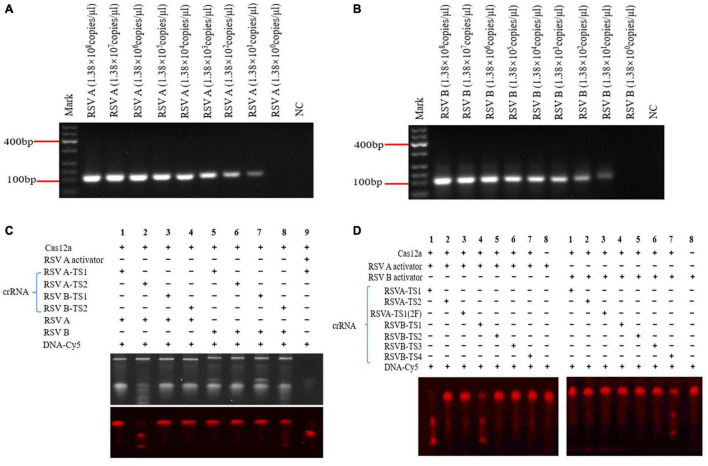
Recombinase polymerase amplification (RPA) on RSV A and RSV B sequences and screening for effective crRNAs. RPA RSV A (110 bp) and RSV B (122 bp) products at different pUC57 plasmids concentrations **(A,B)**. Screening effective crRNAs to activate Cas12a to cleave RSV A and RSV B **(C,D)**. RSV A-TS1-2F crRNA: a modified RSV A-TS1 crRNA. DNA-Cy5: a reporter fragment labeled with fluorophore Cy5. Under yellow, white, and violet light irradiation, the target strip on the gel showed red fluorescence (DNA-Cy5), and the small strip below the target strip showed Cas12a cleavage (C bottom and D). NC: negative control (water) **(A,B)**. NC: DNA-Cy5 and water **(C,D)**. Marker: 600 bp DNA marker. +, added reagent; – no added reagent. All experiments were repeated three times (*n* = 3).

Recombinase polymerase amplification (RPA) was performed according to TwistAmp Liquid Basic Quick Guide. Briefly, 2.4 μl of each primer at 10 μm concentration was added into the 0.2-ml PCR tubes. A premaster mix (per reaction) was prepared in the following the steps below: 25 μl 2 × Reaction Buffer, 9.2 μl dNTPs, 5 μl 10 × Basic E-mix; the mixture was vortexed and spined briefly. About 2.5 μl of 20 × Core Reaction Mix was added to the premaster mix. About 41.7 μl of master mix was added to primers (Table 1) prepared in tubes (step 1) and mixed with a pipette. About 2.5 μl of 280 mM MgOAc and 1 μl RSV A or RSV B DNA template were added to the tube lids. DNA template and MgOAc should be kept separate in the tube lid prior to spin down. The mixture was spined in MgOAc/template and mixed well (6 × inversions) to start a reaction. The final volumes were based on the 50 μl of reaction mixture. The mixture was then subjected to RPA at 39^°^C for 25 min. Aliquots of RPA products were electrophoresed 30 min on 2% agarose gels containing 2 μl GoldView II Nuclear Staining Dyes and analyzed using a Bio-Rad ChemiDoc image acquisition system and Quantity One (v4.6) software (Figures 2A,B).

### Cas12a Cleavage

According to the activation principle of Cas12a, two crRNAs of RSV A-TS1 and RSV A-TS2 for RSV A were designed, and four crRNAs of RSV B-TS1, RSV B-TS2, RSV B-TS3, and RSV B-TS4 for RSV B were designed using benching (Table 1). Various crRNAs were designed and tested to pick the best performing one. According to the position of target sequence, the activator sequence was synthesized, and the single strand was gradually cooled and annealed at 95^°^C for 5 min to form a double strand that cooperates with crRNA to act together to activate the Cas12a. Cas12a cleavage was carried out following the earlier method (Chen et al., 2018) that made simple modifications. Briefly, the Cas12a cleavage reaction consisted of the following mixture: Lba Cas12a (1 μm), crRNA (1 μm), RSV A RPA product or RSV B RPA product or activator (1 μm), and DNA-Cy5 (500 ng/μl), and DEPC-H_2_O was added to make up the 20 μl volume. Finally, the Cas12a cleavage reaction was carried out at 37^°^C in the dark for 25 min, and then, the reaction mixture was placed in a dry box for 5 min (Figures 2C,D).

### Fluorescence Assay

**FIGURE 3:**
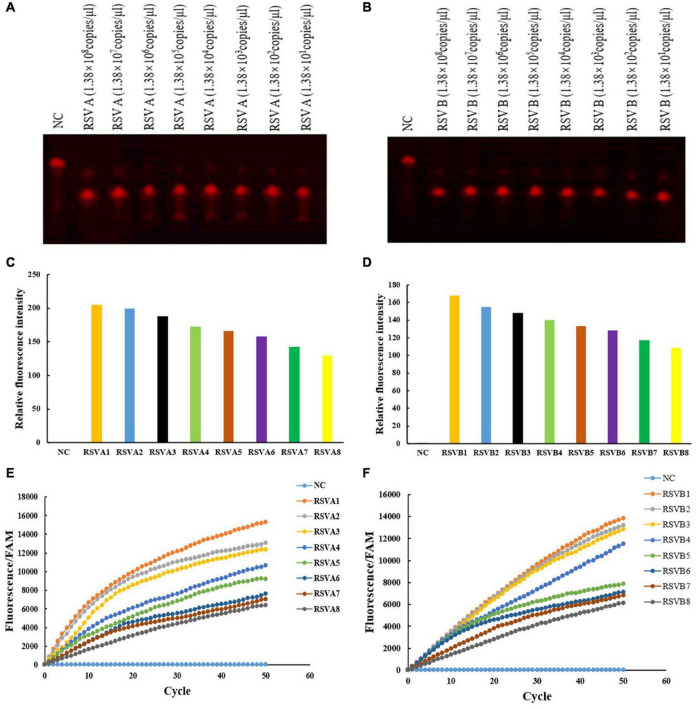
crRNA activated Cas12a to cleave RSV A and RSV B RPA products and the fluorescence detection of the F-Q ssDNA reporter fragment. RSV A-TS2 activated Cas12a to cleave RSV A RPA products using different pUC57 plasmid concentrations **(A)** RSV B-TS4 activated Cas12a to cleave RSV B RPA products using different pUC57 plasmid concentrations **(B)** Fluorescence detection of RSV A and RSV B RPA products after Cas12a cleavage using a fluorescence multimode reader **(C,D)** and a real-time fluorescence PCR machine **(E,F)** RSV A1–RSV A8: 1.38 × 10^8^ copies/μl–1.38 × 10^1^ copies/μl, diluted 10-folds in ddH_2_O each time. RSV B1–RSV B8: 1.38 × 10^8^ copies/μl–1.38 × 10^1^ copies/μl, diluted 10-folds in ddH_2_O each time. NC: DNA-Cy5 and water **(A,B)**. NC: FAM and water **(C–F)**. All experiments were repeated three times (*n* = 3).

For the fluorescence assay of Cas12a cleavage reaction, as described above, we prepared a Cas12a reaction device with 5′-FAM and 3′-TAMRA labeled ssDNA reporter (Table 1; Mukama et al., 2020). Briefly, the Cas12a cleavage reaction consists of the following mixture: Lba Cas12a (1 μm), crRNA (1 μm), RSV A or RSV B RPA product (1 μm), 2 μl 10 × NEB buffer, and 1 μl F-Q ssDNA (Fluorescence-Quenching ssDNA), and DEPC-H_2_O was added to make up the 20 μl volume. The reaction mixture was incubated at 39^°^C for 5 min and then read using a fluorescence multimode reader (Thermo Fisher Scientific, Waltham, MA, United States). The fluorescence reporter from the cleavage was measured (excitation at 495 nm and emission at 521 nm; Figures 3C,D). A real-time fluorescence PCR machine detection could observe the real-time enzyme digestion process, and the fluorescence signal gradually appeared as cycles progressed (Figures 3E,F). Therefore, the Cas12a cleavage reaction was also detected using a real-time fluorescence PCR machine. The reaction mixture was preheated at 39^°^C for 2 min. The temperature did not change. The cycle was used to represent the time point, and the fluorescence signal was read every 20 s, a total of 50 cycles.

### IT-RAISE System

**FIGURE 4:**
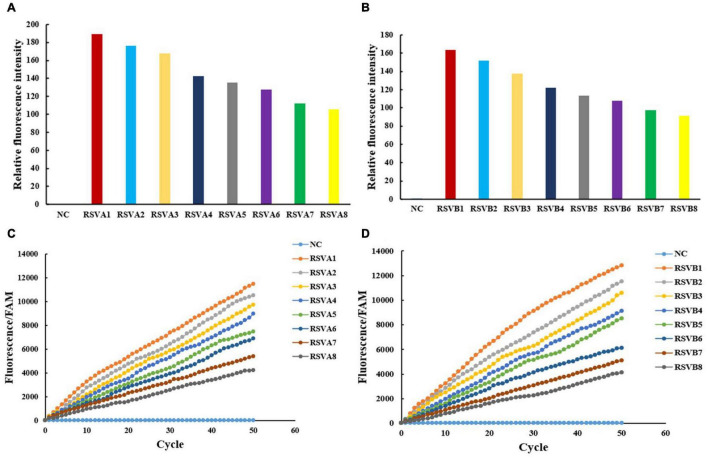
The IT-RAISE system detects RSV A and RSV B. Results were checked using a fluorescence multimode reader **(A,B)** and a real-time fluorescence PCR machine **(C,D)**. Information of RSV A1–RSV A8 and RSV B1–RSV B8 is the same as in Figure 3. NC: FAM and water. All experiments were repeated three times (*n* = 3).

The IT-RAISE system was used to detect RSV A or RSV B, the RSV A or RSV B fragments integrated into pUC57 plasmid concentration of 1.38 × 10^1^–1.38 × 10^8^ copies/μl. The IT-RAISE system was prepared as Component RPA, CRISPR/Cas12a, and fluorescence. Component RPA consisted of 1 μl RSV A or RSV B RPA-F (10 μm), 1 μl RSV A or RSV B RPA-R (10 μm), 12.5 μl 2 × Reaction Buffer, 2 μl dNTPs, 2.5 μl 10 × Basic E-mix, 1.25 μl 280 mM MgOAc, and 0.5 μl RSV A or RSV B DNA template (or the clinical DNA specimens to be tested). Component CRISPR/Cas12a contained the Cas12a-crRNA mix with 1 μl RSV A or RSV B crRNA and 1 μl Lba Cas12a. Component fluorescence consisted of 1 μl of F-Q ssDNA reporters. The final volumes were based on the 25 μl reaction mixture. The reaction mixture was preheated at 39^°^C for 10 min; then, the results were also detected using a fluorescence multimode reader (MULTISKAN FC, Thermo Fisher Scientific, Waltham, MA, United States) and a real-time fluorescence PCR machine (MA-6000, Suzhou Molarray Co. Ltd., Suzhou, China; Figure 4). In a real-time fluorescence PCR machine detection, the fluorescence signal was read every 20 s at 39^°^C, a total of 50 cycles. It should be noted that 280 mM MgOAc reagent needs to be added finally. Under the constant temperature condition of 39^°^C, the 280 mM MgOAc was activated and indicated the start of IT-RAISE system. All reagents were mixed together from the beginning. The total reaction time was 35 min.

### Oropharyngeal Swab Specimen Collection

This study was approved by the Ethics Committee of the Third Affiliated Hospital of Zunyi Medical University (approval no. 2020-049). A total of 125 respiratory tract infection participants were enrolled. The patients were hospitalized in the Department of Pediatric of the Third Affiliated Hospital of Zunyi Medical University from November 2020 to February 2021. The hospitalized children (0–10 years old) were enrolled in the study, whose parents or legal guardians provided written informed consent before the study was started. This study was conducted according to the Declaration of Helsinki-1964. Oropharyngeal swabs were collected using a disposable sampler with a virus preservation solution (COPAN flocked swab) according to the standard protocols, and specific methods and steps are reported in our team’s published articles (Gong et al., 2021). The tip of the swab was placed in the tube with 3 ml of virus preservation solution (Gong et al., 2021). The virus preservation tubes must be tightened and were transferred quickly to the laboratory for storage at −80^°^C.

### RNA Isolation From Oropharyngeal Swab Specimens and Reverse Transcription

We used virus RNA extraction kit to extract RNA from oropharyngeal swab specimens (only viral RNA was extracted, and all the DNA, proteins, and mRNA were removed). A mixture was added in the following the steps below: 200 μl of virus preservation solution, 50 μl virus RNA LB1, and 20 μl protease K; the mixture was vortexed and then digested at room temperature for 5 min. After the digestion, 700 μl virus RNABB2 was added, and then, the mixture was vortexed for 1 min. All the above mixture was poured into the adsorption column and centrifuged at 13,000 rpm for 1 min. The waste liquid was poured out. Then, 500 μl washing buffer was added to the adsorption column and centrifuged at 13,000 rpm for 15 s. the above step was repeated again. The adsorption column was centrifuged at 13,000 rpm for 1 min. The residual liquid was dried thoroughly. The adsorption column core has been put into the 1.5-ml RNase-free collection pipe. About 30–80 μl nuclease-free collection was added to the adsorption column core. Then, the adsorption column core was centrifuged at 13,000 rpm for 1 min. The eluent was viral RNA. Subsequently, the viral RNA was reverse-transcribed to cDNA with the Takara Primescript RT Master Mix.

### Cell Culture, Respiratory Syncytial Virus Infection, and PCR Operation

All the operations were carried out following our previous published procedures (Liu et al., 2018).

### Statistical Analysis

The data are expressed as mean ± standard deviation (SD), *n* = 3. The *t*-test was used for the data between the two groups with normal distribution. One-way ANOVA was used for the comparison among multiple groups. Receiver operating characteristic curves (ROCs) were used to calculate specificity and sensitivity. The data were analyzed using SPSS statistical software (version 23.0, SPSS Inc., Chicago, IL, United States), and *p* < 0.05 was considered as statistically significant.

## Results

### Recombinase Polymerase Amplification on RSV A and RSV B Sequences and Screening Effective crRNAs for Cas12a Activation

Specific RSV A and RSV B DNA sequences integrated into pUC57 plasmids were gradually diluted 10-folds. Then, after RPA, products were detected using 2% agarose gel electrophoresis and sent for Sanger sequencing (BGI, China). Both RPA products were RSV A and RSV B sequences, and a T-rich protospacer adjacent motif (TTTN) was recognized by Cas12a (Supplementary Figures 2A–G). When pUC57 plasmids were diluted to 1.38 × 10^1^ copies/μl, RSV A and RSV B sequences were detected by RPA (Figures 3A,B); RPA detection decreased concomitant with decreased target concentrations. Therefore, RPA exhibited a high amplification efficiency.

Using RSV A and RSV B RPA products, we screened four crRNAs (RSV A-TS1, RSV A-TS2, RSV B-TS1, and RSV B-TS2) that could cleave RSV A and RSV B *via* Cas12a activity (Figures 2C,D). Activators were synthetic fragments according to the target position that could be cleaved by Cas12a and similar functions to the RPA products of RSV in this experiment, which acted as a positive control. The positive control showed Cas12a cleavage functioned normally (Figure 2C, lane 9). RSV A-TS2 showed a good activation of the RSV A RPA product in activating Cas12a (Figure 2C, lane 2), but RSV A-TS1 did not have the same effect (Figure 2C, lane 1). Therefore, we selected RSV A-TS2 as an effective crRNA for Cas12a to cleave RSV A.

RSV B-TS1 also activated Cas12a to cleave RSV A, which suggests the crRNA had overlapping effects (Figure 2D, left lane 4). RSV B-TS1 had no effect on the RSV B RPA product in activating Cas12a (Figure 2C, lane 7). RSV B-TS2 crRNA displayed activation, but the effects were not very good (Figure 2C, lane 8). Therefore, we redesigned both RSV B crRNAs (RSV B-TS3 and RSV B-TS4) for screening (Figure 2D).

RSV B-TS3 had no effect on RSV B activator in activating Cas12a (Figure 2D, right lane 6). RSV B-TS4 had good effect on RSV B activator in activating Cas12a (Figure 2D, right lane 7). Therefore, we selected RSV B-TS4 as an effective crRNA for Cas12a in cleaving RSV B. RSV A-TS1-2F was a modified RSV A-TS1, but the sequences were the same. This modification involved changing the OH at the 2′ position of C and U of RNA to fluorine (F), which was resisted RNase degradation. RSV A-TS1-2F was tested for Cas12a activation; if activation occurred, degradation would not be an issue in later experiments. Unfortunately, it did not activate Cas12a (Figure 2D, left lane 3). Therefore, to ensure the system worked normally, a modified crRNA could not be selected for subsequent experiments. Therefore, our data showed that RSV A-TS2 activated Cas12a to cleave RSV A sequences, but it had no effect on RSV B sequences. RSV B-TS4 activated Cas12a to cleave RSV B sequences, but it had no effect on RSV A sequences, thereby effectively distinguishing RSV A from RSV B. Thus, RSV A-TS2 and RSV B-TS4 exhibited good specificity.

### Effective crRNAs Activate Cas12a to Cleave RSV A and RSV B RPA Products and the Fluorescence Detection of F–Q ssDNA Reporter Fragment

Using RSV A and RSV B RPA products at different pUC57 plasmid concentrations, we verified crRNA activation of RSV A-TS2 and RSV B-TS4; they effectively activated Cas12a to perform cleavage, respectively (Figures 3A,B). Then, a fluorescence multimode reader was used to detect the F–Q ssDNA reporter fragment. The negative control displayed no fluorescence, but RSV A and RSV B RPA products after Cas12a cleavage displayed fluorescence (Figures 3C,D). Also, using a real-time fluorescence PCR machine, RSV A and RSV B RPA product fluorescence after Cas12a cleavage gradually appeared as cycles progressed (Figures 3E,F). Thus, RSV A or RSV B and RSV A-TS2 activated Cas12a to cleave RSV A, and RSV B-TS4 activated Cas12a to cleave RSV B; therefore, fluorescent quenching was terminated and fluorescence appeared (Figure 3). The negative control had no target sequence; it underwent fluorescence quenching, and thus, no fluorescence appeared. Moreover, fluorescence detection decreased with decreasing target sequence concentrations.

### The IT-RAISE System Detects RSV A or RSV B

The RSV A and RSV B were both detected with high amplification efficiency and specificity using a step-by-step approach. However, as a previous study reported that an all-in-one dual CRISPR assay could rapidly detect SARS-CoV-2 (Ding et al., 2020), we wanted to see whether our all-in-one RPA and CRISPR assay systems were equally effective in detecting RSV A and RSV B. In our IT-RAISE system, all RPA components, CRISPR/Cas12a cleavage, and fluorescence detection components were thoroughly mixed in a one-pot reaction system and incubated at 39°C, thereby eliminating the requirement for separate RPA, Cas12a cleavage, and fluorescence detection. Using a fluorescence multimode reader, the negative control displayed no fluorescence, but RSV A and RSV B displayed it (Figures 4A,B). Also, using a real-time fluorescence PCR machine, RSV A and RSV B, fluorescence after IT-RAISE gradually appeared as cycles progressed (Figures 4C,D). Moreover, fluorescence detection decreased with decreasing target sequence concentrations.

### Assessing the IT-RAISE System in RSV A-Infected BEAS-2B Cells

**FIGURE 5:**
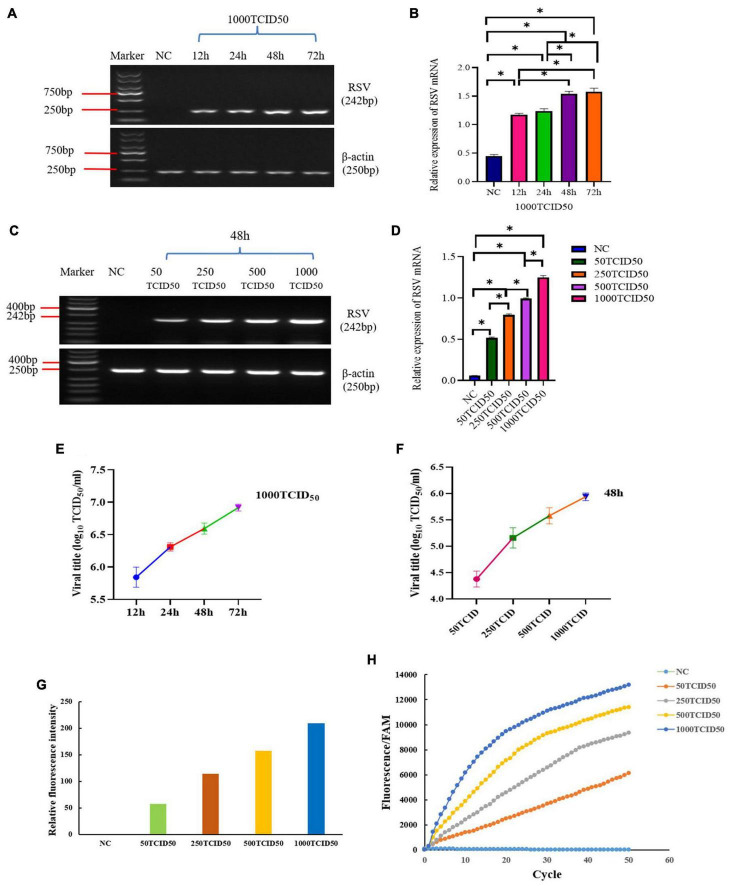
The IT-RAISE system in RSV A-infected BEAS-2B cells. Cells were infected with 1000 TCID50 RSV A at 12h, 24h, 48h and 72h and detect RSV by PCR **(A,B)**. Cells were infected with 50TCID50, 250TCID50, 500TCID50, and 1000TCID50 RSV A at 48 h and detect RSV by PCR **(C,D)**, and afterward RSV titer **(E)** BEAS-2B cells were infected with 50TCID50, 250TCID50, 500TCID50, and 1,000TCID50 RSV A at 48 h, and afterward RSV titer **(F)** The IT-RAISE system detected fluorescence using a fluorescence multimode reader **(G)** and a real-time fluorescence PCR machine **(H)** when BEAS-2B cells were infected with 50TCID50, 250TCID50, 500TCID50, and 1,000TCID50 RSV A at 48 h. Detection of viral DNA using the IT-RAISE system. *p < 0.05 indicates a statistically significant difference. All experiments were repeated three times (*n* = 3).

We assessed the detection capability of the IT-RAISE system in RSV A-infected BEAS-2B cells. Due to the COVID-19 pandemic, an RSV B strain could not be purchased. However, from the previous research of our team (Liu et al., 2018), we selected the RSV A 1,000 median tissue culture infectious dose (TCID50) strain to infect BEAS-2B cells and detect RSV load to determine optimal infection times by PCR experiment (Figures 5A,B). We observed that 48 h was the best infection time. Then, RSV load after infection was observed using different TCID50 values of RSV A at 48 h by PCR experiment. We observed that RSV load increased with increasing RSV infection concentrations (Figures 5C,D). The same result was observed in virus titer detection experiments (Figures 5E,F). Then, BEAS-2B cells were infected with different RSV A TCID50 values for 48 h. Not only we identified the detection capability of the IT-RAISE system, but also the detection results were dependent on the infection concentration. The results from a fluorescence multimode reader and a real-time fluorescence PCR machine showed that fluorescence intensity increased with increased RSV A infection concentration (Figures 5G,H). When combined with other results (Figures 2–5), the IT-RAISE system was robust and could be used for clinical specimens.

### The IT-RAISE System for Clinical Specimens

**FIGURE 6:**
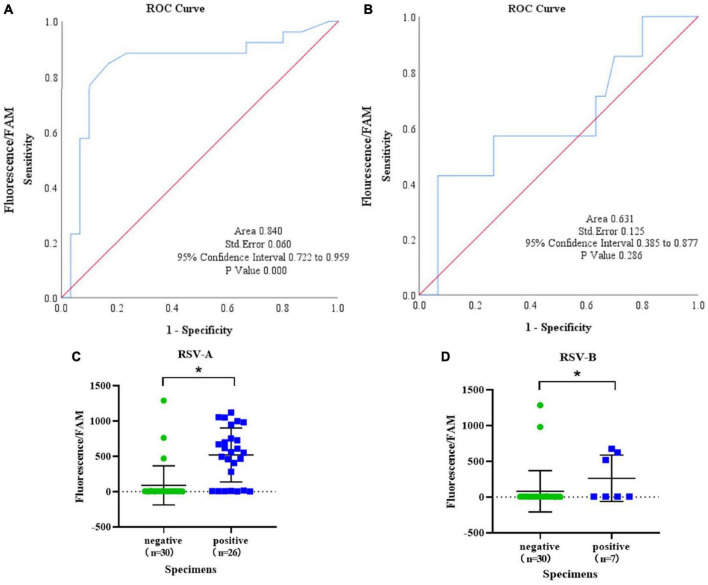
The IT-RAISE system detects RSV in clinical specimens. A ROC curve and scatter diagram for RSV A detection were generated using IT-RAISE system **(A,C)**. A ROC curve and scatter diagram for RSV B detection were generated using IT-RAISE system **(B,D)**. *p < 0.05 indicates a statistically significant difference. All experiments were repeated three times (*n* = 3).

To explore the detection capability of the IT-RAISE system for clinical specimens, we collected 125 oropharyngeal swab specimens from hospitalized children. To increase RSV-positive detection rates, 125 serum RSV IgG-M-positive cases of oropharyngeal swab specimens were first selected and tested using an RSV nucleic acid detection kit and the remaining specimens were screened according to the acquisition sequence. Then, 32 RSV-positive specimens were initially screened (Supplementary Figures 3A–H) and repeat screened (Supplementary Figures 4A,B) to exclude false positives. Then, the 32 specimens underwent ordinary PCR using RSV A and RSV B primers, and samples were sent for Sanger sequencing. Finally, 23 specimens were deemed RSV A positive, four were RSV B positive, three indicated RSV A and RSV B co-infection, and two had no RSV infection (Supplementary Table 1). Next, these 30 RSV-positive specimens were detected using the IT-RAISE system. The results showed 19 RSV A-positive specimens using RSV A-TS2; but no RSV B was found (Supplementary Figure 4C and Supplementary Tables 1, 2). Moreover, we observed 3 RSV B-positive specimens using RSV B-TS4; but no RSV A-positive specimens were found (Supplementary Figure 4D and Supplementary Tables 1, 2). A total of 30 negative control specimens were selected with fluorescence CT values of “−” (means “No CT” or “Negative”) for two times according to the results of RSV nucleic acid detection kit, and then, they were amplified by ordinary PCR using RSV A and RSV B primers and agarose gel electrophoresis still without bands. A total of three false-positive RSV A specimens and two false-positive RSV B specimens were identified by the IT-RAISE system (Figures 6C,D). According to RSV A detection ROC curve analysis, the area under the curve (AUC) was 0.840 [95% confidence interval (CI): 0.722–0.959; Figure 6A]. An RSV A detection scatter diagram showed that when compared to the RSV A-negative control group, fluorescence values in the RSV A-positive group increased significantly (Figure 6C; *p* < 0.05). According to ROC curve analyses of RSV B detection, the AUC was 0.631 (95% CI: 0.385–0.877; Figure 6B). An RSV B detection scatter diagram showed that when compared to the RSV B-negative control group, RSV B-positive group fluorescence values increased significantly (Figure 6D; *p* < 0.05). In theory, the IT-RAISE system should be able to detect co-infections, but due to the detection system which is not perfect, RSV A and RSV B co-infection could not be detected. The IT-RAISE system sensitivity for detecting RSV A was 73.08% (19/26, 95% CI: 51.95–86.75%) and specificity was 90% (27/30, 95% CI: 72.32–97.38%; Supplementary Table 3) and those of RSV B was 42.85% (3/7, 95% CI: 11.81–79.76%) and specificity was 93.33% (28/30, 95% CI: 76.49–98.84%; Supplementary Table 3).

We also calculated RSV A or RSV B detection costs using the IT-RAISE system; the cost was $ 2.6 per test (Supplementary Table 4). However, rapid RNA extraction and RT costs required an additional about $ 2.85 per test. Also, it may be cheaper to buy reagents in the large quantities. We also calculated RSV A or RSV B detection times using the IT-RAISE system, that is, adding reagents (2 min) and the real-time fluorescence PCR machine reaction times (50 cycles; 35 min), thus 37 min/detection assay. Also, rapid viral RNA extraction using centrifugal columns and RT assays required an additional 30 min.

## Discussion

Globally, more than 95% of children under the age of 2 years have been infected with RSV. Bronchiolitis is caused by RSV infection and is an important factor, which leads to increased mortality in these children, with an estimated 99% of deaths occurring in developing countries (Xing and Proesmans, 2019). RSV infection also leads to increased hospitalization and mortality in the elderly and is equivalent to influenza virus infection. Therefore, early and rapid RSV infection diagnostics could have major clinical significance for the early treatment and the prevention of RSV infections and associated complications.

In step-by-step RPA-CRISPR/Cas12a-based fluorescence assays, RPA efficiently amplified RSV A and RSV B DNA sequences at 1.38 × 10^1^ copies/μl. Importantly, we effectively distinguished between RSV A or RSV B sequences using different crRNAs (RSV A-TS2 and RSV B-TS4) to activate Cas12a to specifically cleave sequences. When compared to other detection methods distinguishing these viruses (Nam and Ison, 2019), the total time of step-by-step experiments of RPA-CRISPR/Cas12a system-based fluorescence assay is about 65 min (25 min for RPA, 30 min for Cas12a cleavage, and 5–10 min for fluorescence assay), which saved a lot of time.

To save more time and simplify operation processes (Wang et al., 2020; Sun et al., 2021), we integrated RPA, CRISPR/Cas12a, and fluorescence assay technologies to explore whether integration generated good effects. Interestingly, the developed IT-RAISE system took only 37 min to detect RSV A and RSV B. Additionally, IT-RAISE sensitivity and specificity levels for RSV A were 73.08 and 90%, respectively, and for RSV B, 42.86 and 93.33%, respectively. IT-RAISE system costs were approximately $ 2.6 per RSV A or RSV B assay (excluding rapid RNA extraction and reverse transcription costs). Also, our IT-RAISE system could have other applications detecting other viral or bacterial infections.

There are some limitations to this study. First, our detection method needs two separate steps (RT of the RNA and IT-RAISE system). Second, because the system used different RSV A and RSV B crRNAs, specimens were divided into two tubes for detection. In future work, to simplify operation processes and reduce detection times and costs, our aim is to detect RSV A and RSV B in the same tube. Next, we need to mix the all reagents together of IT-RAISE system (except 280 mM MgOAc) and then sub-assemble them into many small tubes to save the time and reduce errors of adding reagent. Third, we developed a detection method that quickly and efficiently distinguished RSV A or RSV B infection; however, RSV A and RSV B co-infection specimens were not detected. In the future, the system will be optimized to detect RSV A and RSV B co-infections.

In conclusion, the IT-RAISE system displayed good clinical application prospects for detecting RSV A or RSV B infections; it was simple, rapid, and inexpensive and displayed high amplification efficiency, high sensitivity, and high specificity.

## Data Availability Statement

The original contributions presented in the study are included in the article/Supplementary Material, further inquiries can be directed to the corresponding author.

## Ethics Statement

The studies involving human participants were reviewed and approved by the Ethics Committee of The Third Affiliated Hospital of Zunyi Medical University (Approval No. 2020-049, date October 2020). Written informed consent to participate in this study was provided by the participants’ legal guardian/next of kin.

## Author Contributions

DL directed the program. DL and LG acquired funding. LG and XW wrote the manuscript. LG, CW, and ZL performed the experiments. GH, WZ, and JN processed the data. All authors contributed to the article and approved the submitted version.

## Conflict of Interest

The authors declare that the research was conducted in the absence of any commercial or financial relationships that could be construed as a potential conflict of interest.

## Publisher’s Note

All claims expressed in this article are solely those of the authors and do not necessarily represent those of their affiliated organizations, or those of the publisher, the editors and the reviewers. Any product that may be evaluated in this article, or claim that may be made by its manufacturer, is not guaranteed or endorsed by the publisher.
