# Supplementary material for: Integrated Trinity Test With RPA-CRISPR/Cas12a-Fluorescence for Real-Time Detection of Respiratory Syncytial Virus A or B
Source: Front Microbiol. 2022 Mar 31;13:819931. doi: 10.3389/fmicb.2022.819931 (PMC9008541; doi:10.3389/fmicb.2022.819931)
Supplement: Supplementary file 1 [file Table_1.DOCX]

**Supplementary Informations:**

We first investigated specific RSV A and RSV B DNA sequences, established a detection system using RPA, CRISPR/Cas12a, and fluorescence step-by-step assay, and verified the system could detect RSV A or RSV B **(****Supplementary Figure 1A)**. The assay exhibited considerable promise for RSV A or RSV B practical applications. To save time and simplify operating processes, we used one step approach to generate an integrated assay combining RPA-CRISPR/Cas12a-fluorescence technology to detect RSV A or RSV B on the basis of step-by-step experiment **(****Supplementary Figure 1B)**. In essence, all reaction reagents and target DNA sequence were combined and verified.

**
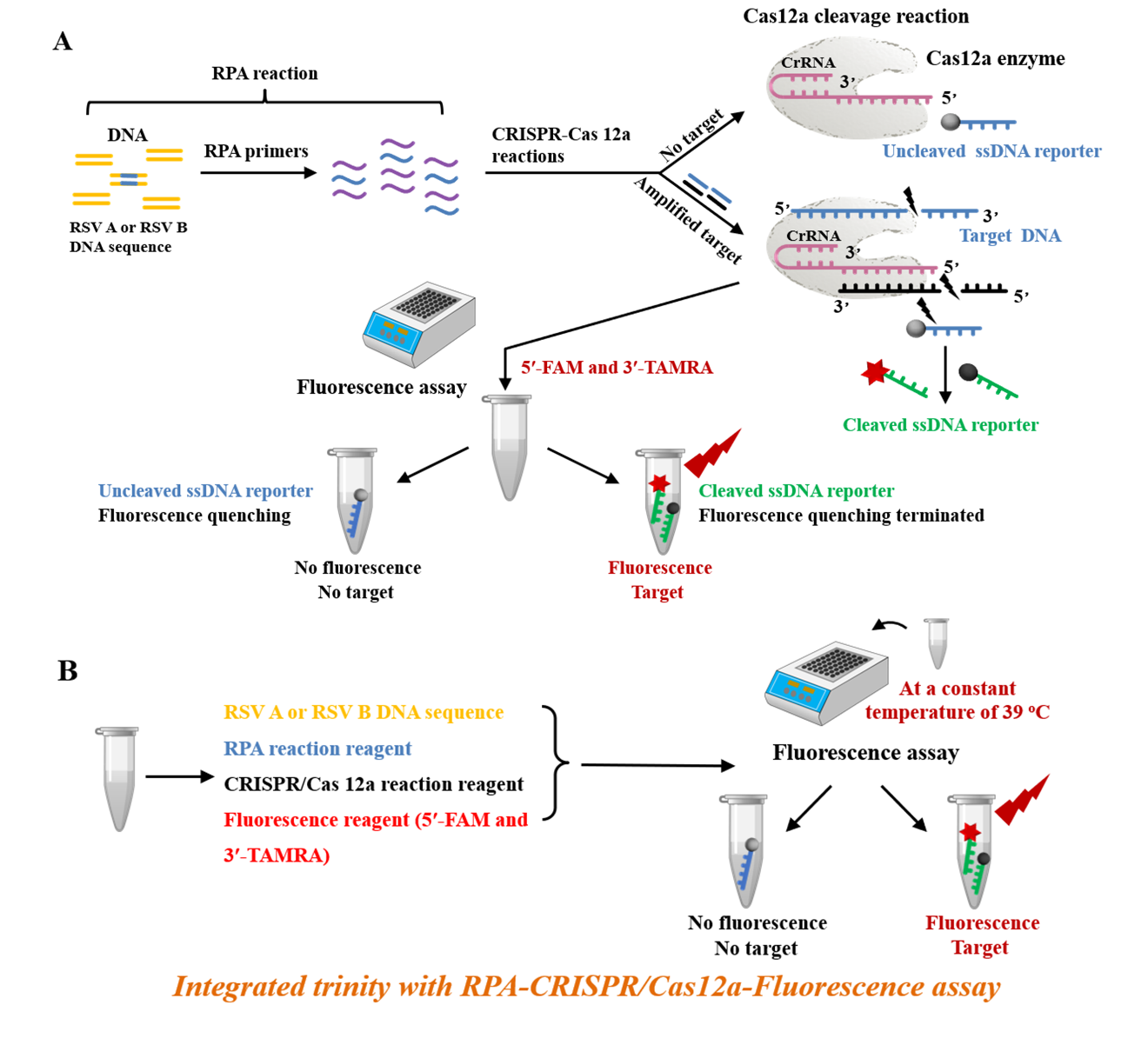
**

SUPPLEMENTARY FIGURE 1. Working principle of the RPA-CRISPR/Cas12a fluorescence assay to detect RSV A or RSV B. Three main steps are involved: RPA assay, Cas12a cleavage, and fluorescence assay. The RPA assay amplifies target DNA to improve detection. If target sequences are present, Cas12a cleaves RPA products and a fluorescent ssDNA reporter (5′-FAM and 3**′**-TAMRA) is generated. If viral sequence are present, they will be cleaved by Cas12a. Fluorescence quenching is terminated and fluorescence occurs. If no viral sequences are present, no fluorescence occurs. **(Supplementary Figure 1A)**.

To determine if RPA products were RSV A or RSV B target sequences and if a T-rich protospacer adjacent motif (TTTN) could be recognized by Cas12a, products were integrated into pUC57 plasmids, diluted to 1.38×10^1^ copies/μL and sent for Sanger sequencing **(Supplementary Figure 2A)**. The RPA products were the same as RSV A and RSV B sequences, and a TTTN sequence present and recognized by Cas12a **(Supplementary Figure 2B–G)**. A TTTN sequence (N represents any base) is shown **(Supplementary Figure 2B)**. RSV A sequencing comparison results; numbers 133–136 are TTTN sequences (**Supplementary** **Figure 2D)**. TTTN sequences (N represents any base) are shown (**Supplementary Figure 2F)**. RSV B sequencing comparison results; numbers 104–107 are TTTN sequences (**Supplementary Figure 2G)**.


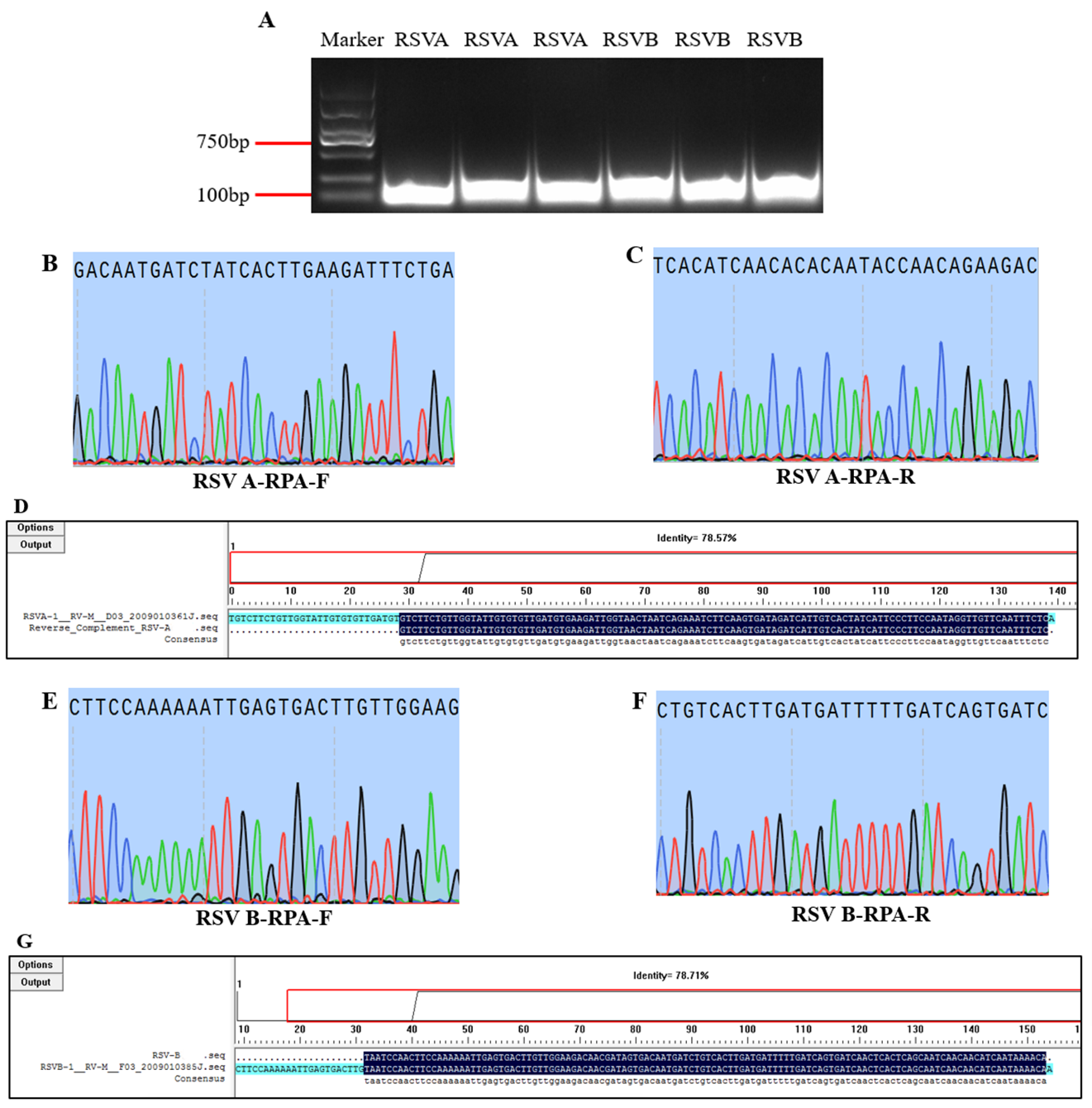


SUPPLEMENTARY FIGURE 2. Sanger sequencing of RSV A and RSV B RPA products. Agarose gel electrophoresis showing RSV A and RSV B RPA products (1.38×10^1^ copies/μL) **(A)**. Sanger sequencing of RSV A RPA products **(B) (C)**. Comparison result of RSV A sequence **(D)**. Sanger sequencing of RSV B RPA products **(E) (F)**. Comparison result of RSV B sequence **(G)**.

To explore IT-RAISE detection capability in clinical specimens, we used 125 oropharyngeal swab specimens from hospitalized children. To increase RSV positive detection rates, serum RSV IgG–M positive from 125 specimens were selected and tested using an RSV nucleic acid detection kit **(Supplementary Figures 3A–H)**, and the remaining specimens were screened according to the acquisition sequence. Finally, 32 RSV positive oropharyngeal swab specimens were screened.


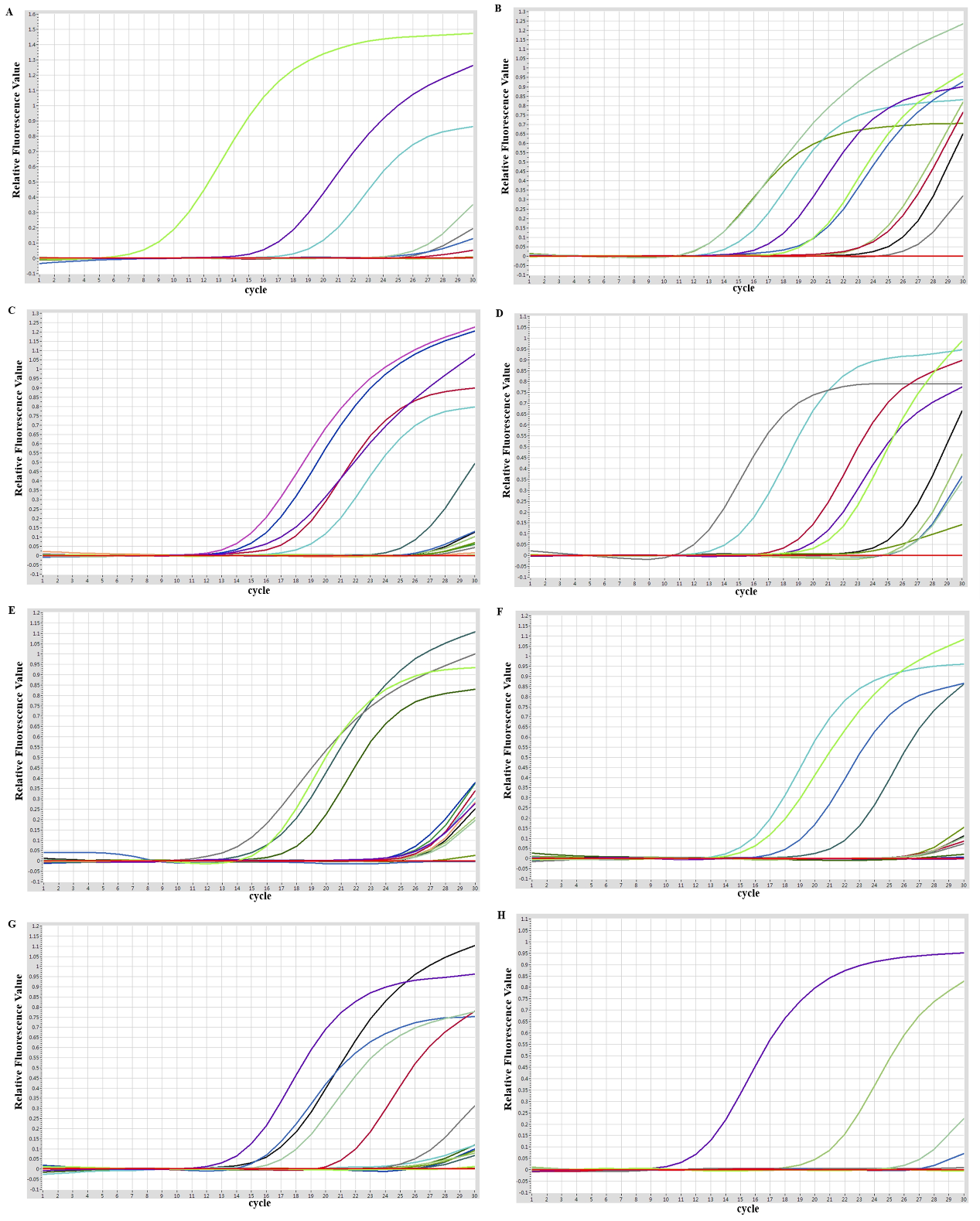


SUPPLEMENTARY FIGURE 3. 32 RSV positive oropharyngeal swab specimens were screened using an RSV nucleic acid detection kit **(A–H)**.

These positive specimens were repeat screened **(Supplementary Figure 4A and Figure 4B)** to exclude false positives. Then, specimens were processed using ordinary PCR and RSV A and RSV B primers and sent for Sanger sequencing. Finally, 23 specimens were deemed RSV A positive, 4 were RSV B positive, 3 were RSV A and RSV B co-infected, and 2 had no RSV infection **(Supplementary Table 1).** Next, these 30 RSV positive specimens were detected by using the IT-RAISE system. The results showed 19 RSV A positive specimens using RSV A-TS2; but no RSV B was found **(Supplementary Figure 4C, Supplementary Tables 1 and 2)**. Moreover, we observed 3 RSV B positive specimens using RSV B-TS4; but no RSV A positive specimens were found **(Supplementary Figure 4D, Supplementary Tables 1 and 2)**. The IT-RAISE system sensitivity for detecting RSV A was 82.61% (19/23, 95% CI: 60.45–94.28%) and specificity was 90% (27/30, 95% CI: 72.32–97.38%) **(Supplementary Table 3)**. The IT-RAISE system sensitivity for detecting RSV B was 75% (3/4, 95% CI: 21.94–98.68%) and specificity was 93.33% (28/30, 95% CI: 76.49–98.84%) **(Supplementary Table 3)**.


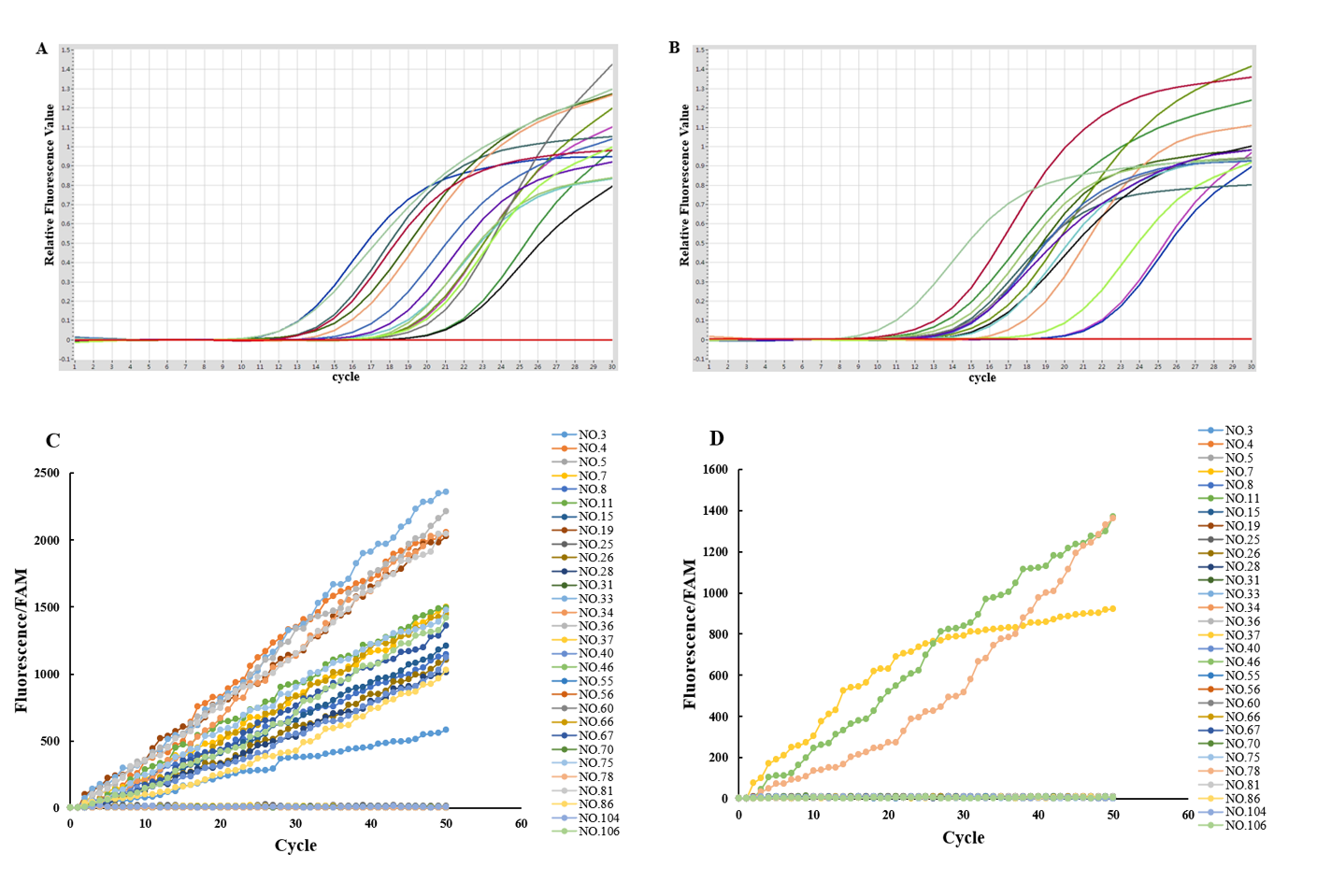


SUPPLEMENTARY FIGURE 4. The IT-RAISE system and clinical specimen detection. RSV positive oropharyngeal swab specimens were screened using an RSV nucleic acid detection kit **(A) (B)**. RSV positive specimens were detected using an IT-RAISE system **(C) (D)**. C and D experiments were repeated three times (n = 3).

**TABLE 1. RSV Positive and Negative Clinical Oropharyngeal Swab Specimens**

| **32 cases of RSV positive (Sanger sequencing)** | | | | **30 cases of RSV negative** |
| --- | --- | --- | --- | --- |
| **RSV A** | **RSV B** | **RSV A and RSV B** | **Not detected** |  |
| NO.3, NO.4, NO.5, NO.7, NO.8, NO.11, NO.15, NO.19, NO.25, NO.26, NO.28, NO.33, NO.34, NO.36, NO.40, NO.66, NO.67, NO.70, NO.75, NO.81, NO.86, NO.104, NO.106 | NO.37, NO.46, NO.55, NO.78 | NO.31, NO.56, NO.60 | NO.1, NO.30 | NO.2, NO.10, NO.12, NO.13, NO.17, NO.18, NO.20, NO.22, NO.23, NO.27, NO.44, NO.54, NO.57, NO.63, NO.72, NO.73, NO.74, NO.76, NO.77, NO.80, NO.83, NO.84, NO.85, NO.90, NO.93, NO.102, NO.105, NO.108, NO.113, NO.122 |

**TABLE 2. Characteristics of Clinical Specimens**

| **Specimens** | | **Collection of**  **specimens (mm-dd-yyyy)** | **Age** | **Sex** | **PCR and**  **Sanger sequencing** | **IT-RAISE system** |
| --- | --- | --- | --- | --- | --- | --- |
| **RSV-positive cases** | NO.3 | 11/22/2020 | 1m6d | F | RSV A | RSV A |
|  | NO.4 | 11/23/2020 | 10m8d | F | RSV A | RSV A |
|  | NO.5 | 11/23/2020 | 1y | F | RSV A | negative |
|  | NO.7 | 11/25/2020 | 8m13d | M | RSV A | RSV A |
|  | NO.8 | 11/25/2020 | 3m17d | F | RSV A | RSV A |
|  | NO.11 | 11/27/2020 | 6m24d | M | RSV A | RSV A |
|  | NO.15 | 11/29/2020 | 9m7d | F | RSV A | RSV A |
|  | NO.19 | 11/29/2020 | 10m23d | M | RSV A | RSV A |
|  | NO.25 | 11/30/2020 | 8m1d | M | RSV A | negative |
|  | NO.26 | 11/30/2020 | 2y9m | F | RSV A | RSV A |
|  | NO.28 | 12/03/2020 | 1y | M | RSV A | RSV A |
|  | NO.31 | 12/06/2020 | 9m2d | M | RSV A and RSV B | negative |
|  | NO.33 | 12/06/2020 | 11m16d | F | RSV A | RSV A |
|  | NO.34 | 12/06/2020 | 9m5d | F | RSV A | RSV A |
|  | NO.36 | 12/06/2020 | 10m17d | M | RSV A | RSV A |
|  | NO.37 | 12/09/2020 | 1y8m | M | RSV B | RSV B |
|  | NO.40 | 12/09/2020 | 9m12d | F | RSV A | RSV A |
|  | NO.46 | 12/30/2020 | 1y6m | F | RSV B | RSV B |
|  | NO.55 | 01/05/2021 | 9m23d | M | RSV B | negative |
|  | NO.56 | 01/05/2021 | 1y2m | F | RSV A and RSV B | negative |
|  | NO.60 | 01/08/2021 | 11m12d | M | RSV A and RSV B | negative |
|  | NO.66 | 01/08/2021 | 2y3m | M | RSV A | RSV A |
|  | NO.67 | 01/12/2021 | 2m24d | F | RSV A | RSV A |
|  | NO.70 | 01/12/2021 | 1y6m | M | RSV A | negative |
|  | NO.75 | 01/12/2021 | 1y | M | RSV A | RSV A |
|  | NO.78 | 01/15/2021 | 2m25d | M | RSV B | RSV B |
|  | NO.81 | 01/16/2021 | 7m29d | M | RSV A | RSV A |
|  | NO.86 | 01/17/2021 | 7m14d | M | RSV A | RSV A |
|  | NO.104 | 01/27/2021 | 1y3m | M | RSV A | negative |
|  | NO.106 | 01/27/2021 | 1y3m | F | RSV A | RSV A |
| **RSV-negative cases** | NO.2 | 11/22/2020 | 4y1m | M | negative | negative |
|  | NO.10 | 11/27/2020 | 5m27d | M | negative | RSV A |
|  | NO.12 | 11/27/2020 | 1y19m | M | negative | negative |
|  | NO.13 | 11/27/2020 | 1y7m | F | negative | RSV B |
|  | NO.17 | 11/30/2020 | 6m12d | M | negative | RSV A |
|  | NO.18 | 11/30/2020 | 6m16d | M | negative | negative |
|  | NO.20 | 11/30/2020 | 8y7d | M | negative | RSV B |
|  | NO.22 | 11/30/2020 | 1y7m | F | negative | negative |
|  | NO.23 | 11/30/2020 | 6m1d | M | negative | negative |
|  | NO.27 | 12/03/2020 | 3m18d | M | negative | negative |
|  | NO.44 | 12/29/2020 | 5m6d | M | negative | negative |
|  | NO.54 | 01/05/2021 | 1m | F | negative | negative |
|  | NO.57 | 01/07/2021 | 2y2m | F | negative | negative |
|  | NO.63 | 01/08/2021 | 1y4m | M | negative | negative |
|  | NO.72 | 01/12/2021 | 1y4m | M | negative | negative |
|  | NO.73 | 01/13/2021 | 7m13d | F | negative | negative |
|  | NO.74 | 01/13/2021 | 3m25d | M | negative | negative |
|  | NO.76 | 01/13/2021 | 3m16d | M | negative | negative |
|  | NO.77 | 01/15/2021 | 1y2m | M | negative | negative |
|  | NO.80 | 01/16/2021 | 8m16d | F | negative | negative |
|  | NO.83 | 01/16/2021 | 2y3m | F | negative | negative |
|  | NO.84 | 01/17/2021 | 11m20d | M | negative | negative |
|  | NO.85 | 01/17/2021 | 3m8d | M | negative | RSV A |
|  | NO.90 | 01/17/2021 | 11m22d | M | negative | negative |
|  | NO.93 | 01/20/2021 | 3y4m | F | negative | negative |
|  | NO.102 | 01/27/2021 | 2m2d | F | negative | negative |
|  | NO.105 | 01/27/2021 | 10m1d | F | negative | negative |
|  | NO.108 | 01/28/2021 | 1y2m | F | negative | negative |
|  | NO.113 | 01/29/2021 | 1y4m | M | negative | negative |
|  | NO.122 | 02/07/2021 | 1y11m | M | negative | negative |

NO.:number; y: year; m: month; d: day

**TABLE 3. Clinical validation of RSV A and RSV B infection**

| **Methods** | **Test content** | **Performance** | **%** | **95% CI** |
| --- | --- | --- | --- | --- |
| **IT-RAISE system** | RSV A | Sensitivity | 73.08% (19/26) | 51.95% - 87.65% |
|  |  | Specificity | 90% (27/30) | 72.32% - 97.38% |
|  | RSV B | Sensitivity | 42.86% (3/7) | 11.81% - 79.76% |
|  |  | Specificity | 93.33% (28/30) | 76.49% - 98.84% |

We also calculated RSV A or RSV B detection costs using the IT-RAISE system; the cost was approximately $ 2.6 per test **(Supplementary Table 4)**.

**TABLE 4. Cost of IT-RAISE System**

| **Materials** | **Specifications** | **Cost** | **Cost of per tset** |
| --- | --- | --- | --- |
| TwistAmp ™ Liquid Basic kit | 200 times/kit | $ 430 | $ 2.15 |
| EnGen^®^Lba Cas12a enzyme | 2,000 pmoles | $ 461 | $ 0.15 |
| RSV A-TS2 or RSV B-TS4 | 50 uL (100μM) | $ 130 | $ 0.3 |
| RSV A or RSV B-RPA primer | 20 OD | $ 15.4 | $ 0.015 |
| dNTPS | 1 mL | $ 3.1 | $ 0.015 |
| F-Q ssDNA | 50 uL (100μM) | $ 80 | $ 0.015 |
| Total cost |  |  | $ 2.645 |
